# Long-range connections are crucial for synchronization transition in a computational model of Drosophila brain dynamics

**DOI:** 10.1038/s41598-022-17544-x

**Published:** 2022-11-22

**Authors:** Shuihan Qiu, Kaijia Sun, Zengru Di

**Affiliations:** 1 International Academic Center of Complex Systems, Beijing Normal University, Zhuhai, 519087 China; 2 School of Systems Science, Beijing Normal University, Beijing, 100875 China

**Keywords:** Biophysics, Computational biophysics, Biological physics, Statistical physics, thermodynamics and nonlinear dynamics

## Abstract

The synchronization transition type has been the focus of attention in recent years because it is associated with many functional characteristics of the brain. In this paper, the synchronization transition in neural networks with sleep-related biological drives in Drosophila is investigated. An electrical synaptic neural network is established to research the difference between the synchronization transition of the network during sleep and wake, in which neurons regularly spike during sleep and chaotically spike during wake. The synchronization transition curves are calculated mainly using the global instantaneous order parameters *S*. The underlying mechanisms and types of synchronization transition during sleep are different from those during wake. During sleep, regardless of the network structure, a frustrated (discontinuous) transition can be observed. Moreover, the phenomenon of quasi periodic partial synchronization is observed in ring-shaped regular network with and without random long-range connections. As the network becomes dense, the synchronization of the network only needs to slightly increase the coupling strength *g*. While during wake, the synchronization transition of the neural network is very dependent on the network structure, and three mechanisms of synchronization transition have emerged: discontinuous synchronization (explosive synchronization and frustrated synchronization), and continuous synchronization. The random long-range connections is the main topological factor that plays an important role in the resulting synchronization transition. Furthermore, similarities and differences are found by comparing synchronization transition research for the Hodgkin-Huxley neural network in the beta-band and gammma-band, which can further improve the synchronization phase transition research of biologically motivated neural networks. A complete research framework can also be used to study coupled nervous systems, which can be extended to general coupled dynamic systems.

## Introduction

Synchronization, as one of the most important collective dynamical behavior of the neural networks, has aroused extensive attention due to its great theoretical significance and potential applications in physical and biological oscillatory systems^1–3^. A great deal of meaningful works about synchronization have been widely reported in many living organisms^4–9^. Particularly, neural synchronization has a basic role in brain functions such as sleep, memory, vision, perception, thought, etc. Generally speaking, most research results indicate that the occurrence of neuronal synchronization is determined by synaptic interaction dynamics, external inputs, the interplay between the intrinsic properties of individual neurons, and network organization features.

In neural systems, it is widely believed that the origin of oscillatory brain rhythms is synchronization. The findings from theoretical studies suggest that synchronization in a neuronal network occurs as one increases synaptic strength. How the transition occurs is crucial. Synchronization must occur at a moderate level in a normal brain because deficit of synchronization leads to autism and schizophrenia, while Parkinson or epilepsy are related to excessive synchronization^10^. Hence, it is believed that normal brain activity is thought to be functioning at a critical point between order and randomness. From this perspective, a slight increase in neural interactions may cause a synchronization transition in local neural circuits. For instance, the synchronization transition can be categorized into two types: continuous and discontinuous. Continuous means that a small change will cause small changes in the system response, and discontinuous means that a small change will cause dramatic changes in the system response. Therefore, investigations of the synchronization transition are very valuable and meaningful. Recently, many meaningful advances in synchronization transition have been reported^11–16^. Khoshkhou and Montakhab^13^ investigated the influence of synaptic interaction (chemical and electrical) as well as structural connectivity on beta-band synchronization transition in network models of lzhikevich neurons, and these results indicated that biologically meaningful models of neural dynamics show a synchronization transition that relies on the average firing frequency of neurons, which is contrary to the case of simple phase oscillators. The authors also provided a systematic study of gamma-band synchronization in spiking Hodgkin-Huxley neurons which interact via electrical or chemical synapses, and concluded that gamma-rhythms are distinctly different from beta-rhythms in^14^. Boaretto et al.^16^ explored the non-monotonic synchronization transition in a Huber-Braun model, and showed that this non-monotonic phase transition occurs due to an interplay between the individual-regular behavior of the neurons and the influence of the synaptic current.

We spend one third of our life asleep, however, how the synchronization transition between neurons changes during sleep and wake is still not clear. Brain oscillations are usually divided into different frequency bands^17^. Especially, alpha rhythms (8–12 Hz) are associated with beginning or end of the sleep and are often intermittent. Although alpha-band oscillations have a significant role in brain functions, they have attracted less attention than other frequency bands^18–20^. Klimesch^18^ considered the relation between alpha-band oscillations and attention, and obtained corresponding results that alpha-band oscillations have two functions: timing and inhibition which are closely related to two fundamental roles of attention: selection and suppression. Moreover, alpha-band oscillations may reflect the most basic cognitive processes and have also been shown to play a key role in the coalescence of brain activity at different frequencies. In^20^, Yap et al. observed the activity of the brain of Drosophila and applied several experimental methods, such as heating, injecting drugs (gaboxadol), genetic activation and so on, to induce sleep. The brain activity of Drosophila during induced and spontaneous sleep was compared by performing local field potential recordings and a transitional sleep stage associated with an approximately 8 Hz oscillation was found in the central brain during spontaneous sleep. Unfortunately, very few research results on the study of synchronization transition in neural networks with sleep-related biological drives have been reported^21^. Hopkins et al. constructed a minimal model of four coupled conductance-based neurons and considered the spikes synchronization of two gap junction-coupled thalamic neurons in^21^. Hence, the synchronization transition of neural networks during sleep and wake requires further investigation.

**Figure 1:**
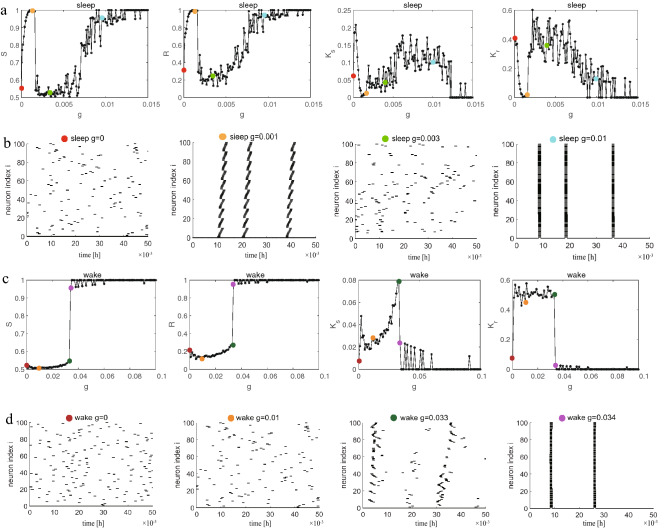
Synchronization diagrams of the coupled neurons in lattice network with ($k=4$) during sleep and wake. (**a**) The global order parameter *S*, *R* and the corresponding susceptibility $\kappa _{S}$, $\kappa _{r}$ during sleep. (**b**) Raster plots corresponds to four specially colored points in (**a**). (**c**) The global order parameter *S*, *R* and the corresponding susceptibility $\kappa _{S}$, $\kappa _{r}$ during wake. (**d**) Raster plots corresponds to four specially colored points in (**c**).

Network connectivity not only has a great influence on collective behavior patterns, especially synchronization^26,27^, but is also a key factor in determining the order of synchronization transition. The network connection is composed of synaptic connections between neurons. In this study, we use the experimentally documented synaptic types: gap junctions or electrical synapses to describe the functional form of synaptic interactions. The role of electrical synapses depends on their special property-bidirectional property, which allows them to coordinate the activities of a large number of interconnected neurons. Based on this bidirectional and coupling nature, electrical synapses play an important role in the synchronization of neural activities and noise reduction^28–31^, especially in regulating sleep. However, the role of the structure of electrical synapses in sleep regulation is poorly understood^32,33^. Therefore, an electrical synaptic neural network is built to study the collective behavior of the network during sleep and wake. First, based on the Huber-Braun thermoreceptor model^22^, we establish a coupled network model that is qualitatively similar to Drosophila sleep. The sleep-wake cycle is adjusted by the circadian rhythm of Drosophila oscillators^23^. The circadian oscillator has positive and negative feedback loops. During sleep, dCLOCK starts to decrease because it needs to activate *per* transcription to synthesize PER, thus the concentration of PER protein is increased. During wake, the PER protein binds dCLOCK and thereby represses *per* transcription, and activates dCLOCK synthesis. dCLOCK begins to accumulate, and the concentration of PER is decreased. Based on Drosophila oscillators (dCLOCK and PER), we designed an external input current $I_{ext_i}=I_{dclocki}-I_{peri}$ that can change the membrane potential *V* according to the different states of sleep and wake. Then, we judged wake or sleep on the basis of the local field potential (LFP). Numerous previous studies have shown that sleep in Drosophila is linked to reduced LFP activity compared to that during wake, moreover, the authors found a transitional sleep stage associated with a 7–10 Hz oscillation during spontaneous sleep in flies. This oscillation was largely absent during wake^20^. The LFP is calculated by the distance-dependent LFP^24^. These results calculated by the model are roughly consistent with the experimental data shown in Fig. 1C, E in the research article by^20^. The above results have been published in^25^. Broadly speaking, the key factors for instance heterogeneity (each node in the network has a different degree), small-world effect(high clustering coefficient and short average path length), clustering (the degree to which friends of a node are also friends), etc. have a fundamental impact on the general collective behavior of a network. Hence, our research begins by focusing on regular networks with no randomness, as well as high clustering but large average path length. We then discuss random networks where each neuron is randomly increased some long-range connections based on the regular network, which have a short average path length but low clustering. Finally, small-world networks are investigated, which have small-world effects including short average path length and high clustering coefficient.

Based on the points discussed above, the principal goals of this paper are to analyse the synchronization transition of the electrical coupled network model with sleep-related biological drives under different network connectivity and further improve synchronization transition research for biologically motivated neural networks. To clearly observe the difference between sleep and wake, we adjusted the sleep-related biological drives to keep neurons only in a sleep or wake state. First, the sleep stage occurs at zero or low current input $I_{ext_i} \in [0,0.3]$ and the wake stage occurs when a strong current is applied ($I_{ext_i} > 2.2$). Then, we use two global instantaneous order parameters *S* and the Kuramoto order parameter *R* as the function of synchronization, and observe the synchronization of the coupled network during wake and sleep by changing the coupling strength. Based on the two different order parameters, qualitatively similar results are obtained. Finally, the role of network topology on synchronization transition is examined, and these structures are divided into three cases: regular networks, the random networks and the small-world networks.

Our main results are as follows: (1) A frustrated synchronization transition (discontinuous transition) can be observed during sleep, in addition, it is independent of the structure and shape of the network. (2) While it is the opposite during wake and three mechanisms of synchronization transition are discovered: explosive synchronization (discontinuous transition), continuous synchronization, and explosive synchronization as well as frustrated synchronization appear simultaneously. The random long-range connections are the main topological factor that play an important role in the resulting synchronization transition. (3) The phenomenon of quasi-periodic partial synchronization can be observed during sleep but is absent during wake in ring-shaped networks. (4) We find that patterns of synchronization for neural networks with sleep-related biological drives in alpha-band are distinctly different from the corresponding transition in HH neural networks in beta-band and gamma-band.

## Models and methods

A single neuron of our networks is modeled based on the Huber-Braun thermoreceptor model^22^ as follows:

1 $$\begin{aligned} C\frac{dV_{i}}{dt}=-I_{l_i}-\alpha (I_{Na_i}+I_{K_i}) -\beta (I_{pNa_i}+I_{K,Ca_i})+I_{gj_i}+I_{ext_i} \end{aligned}$$

where $i=1,2,\cdots ,N$ represents the number of neurons. $V_{i}$ is the membrane potential of the *i*-th neuron. *C* is the membrane capacitance. $I_{l_i}$ is the leakage current of the *i*-th neuron: $I_{l_{i}}=g_{l_{i}}(V_{i}-E_{l})$, where $g_{l_{i}}$ is the conductance and $E_{l}$ is the equilibrium potential. $I_{Na_i}$ and $I_{K_i}$ are the fast depolarizing and repolarizing currents for the spike generation of the *i*-th neuron, respectively. $I_{pNa_i}$ and $I_{K,Ca_i}$ are slow currents for subthreshold oscillations. The role of the parameters $\alpha ,\beta$ is to alter the magnitude of the spike currents or subthreshold currents separately.

The voltage-dependent ionic currents are given in the following equations:

2 $$\begin{aligned} I_{j_{i}}=g_{j_{i}}a_{j_{i}}(V_{i}-V_{j}) \end{aligned}$$

where $$j=Na, K, pNa, K,Ca$$.

$$\begin{aligned}&a_{Na_{i}}=\frac{1}{1+exp[-s_{Na}(V_{i}-V_{0Na})]} \\&\tau _{K}\frac{da_{K_{i}}}{dt}=\frac{1}{1+exp[-s_{K}(V_{i}-V_{0K})]}-a_{K_{i}} \\&\tau _{pNa}\frac{da_{pNa_{i}}}{dt}=\frac{1}{1+exp[-s_{pNa}(V_{i}-V_{0pNa})]}-a_{pNa_{i}}\\&\tau _{K,Ca}\frac{da_{K,Ca_{i}}}{dt}=-\eta I_{pNa_{i}}-{\xi a_{K,Ca_{i}}} \end{aligned}$$

where $\tau _{j}$ is the time delay. $s_{j}$ is the slope of the activation curve. $V_{0j}$ is the half-activation potential. $\eta$ is the coupling constant, and $\xi$ is the relaxation factor.

### Remark 1

In the Huber-Braun thermoreceptor model^22^, the voltage-dependent ionic currents are expressed as $I_{i}=\rho g_{i}a_{i}(V-E_{i})$, the direct coupling of the slow repolarizing current to the slow depolarizing current are expressed as $\dot{a}_{sr}= \phi (\eta I_{sd}-ka_{sr})/\tau _{sr}$, and the temperature dependences are expressed by $\rho$ and $\phi$. $\rho =1.3^{(T-T_0)/\Delta }$, $\phi =3.0^{(T-T_0)/\Delta }$, where °C is the reference temperature. In this manuscript, we focus on the difference between the synchronization transition of the network during sleep and wake and do not consider the effect of temperature on sleep and wake, therefore we set the default temperature $T=25$ °C, and the parameter $\rho =1, \phi =1$.

The current $I_{gj_i}$ is the total synaptic current received by neuron *i*. For a gap junction, the synaptic current is

3 $$\begin{aligned} I_{gj_{i}}=\sum _{nb\in ADJ(i)}g_{gj}(V_{nb}-V_{i}) \end{aligned}$$

where *nb* represents the set of elements linked to the *i*-th neuron. $g_{gj}$ is the coupling strength, and $I_{ext_i}$ is the external input of the *i*-th neuron from sleep-related biological drives used to regulate the sleep-wake cycles.

Sleep-related biological drives include interconnected positive and negative feedback loops^23,35,36^. In this paper, a simplified model that represents the dynamics of the positive and negative feedback loops of the Drosophila oscillator was used^23^. A negative feedback loop is included, in which PER protein represses *per* transcription by binding the dCLOCK transcription factor. A positive feedback loop is also included, in which dCLOCK indirectly enhances its own formation. During sleep, dCLOCK starts to decrease because it needs to activate *per* transcription to synthesize PER, and thus, the concentration of PER protein is increased. During wake, the PER protein binds dCLOCK and thereby represses *per* transcription, and activates dCLOCK synthesis. dCLOCK then begins to accumulate, and the concentration of PER is decreased. Based on Drosophila oscillators, we designed an external input current $I_{ext_i}=I_{dclock_{freei}} =I_{dclocki}-I_{peri}$ that can change the membrane potential $V_{i}$ according to the different states of sleep and wake. The current $I_{dclock_{freei}}$ means the postsynaptic current in neuron *i* from Drosophila oscillator, the sleep stage occurs at zero or low current input $I_{dclock_{freei}} \in [0,0.3]$ and the wake stage occurs when a strong current is applied ($I_{dclock_{freei}} > 2.2$).

$$\begin{aligned}&I_{ext_i}=I_{dclock_{freei}}=I_{dclocki}-I_{peri}\\&I_{dclocki}=g_{dclocki}[dCLOCK](E_{syn}-V_{i})\\&I_{peri}=g_{peri}[PER](E_{syn}-V_{i}) \end{aligned}$$

The differential equations for [dCLOCK] and [PER] are based on an earlier published model of the Drosophila circadian oscillator^23^.

4 $$\begin{aligned}&\frac{d[dCLOCK]}{dt}=v_{sc}R_{sc}-k_{dc}[dCLOCK] \end{aligned}$$

5 $$\begin{aligned}&R_{sc}=<\frac{K_{2}}{K_{2}+[dCLOCK_{free}]}>\tau _{2} \end{aligned}$$

6 $$\begin{aligned}&\frac{d[PER]}{dt}=v_{sp}R_{sp}-k_{dp}[PER] \end{aligned}$$

7 $$\begin{aligned}&R_{sp}=<\frac{[dCLOCK_{free}]}{K_{1}+[dCLOCK_{free}]}>\tau _{1} \end{aligned}$$

where $[dCLOCK_{free}]=[dCLOCK]-[PER]$ or zero, whichever is greater. $\tau _{1}$ denotes the time delay between *per* transcription and the synthesis of new PER protein. $\tau _{2}$ means the time delay between *dclock* transcription and the synthesis of new dCLOCK protein. The models (4)–(7) have been described in detail before^23,37,38^ so here we only provide a brief summary of the unified model and report model parameters in Table 1 for completeness.

**Table 1:** The parameters values for the model (1).

Parameter	Value	Unit	Parameter	Value	Unit
C	1	nF/cm^2^	$g_{l}$	0.4	ms/cm^2^
$V_{l}$	− 60	mV	$g_{Na}$	1.3	ms/cm^2^
$V_{Na}$	50	mV	$g_{K}$	1.75	ms/cm^2^
$V_{K}$	− 90	mV	$g_{pNa}$	0.22	ms/cm^2^
$V_{0Na}=V_{0K}$	− 25	*mV*	$g_{K,Ca}$	0.35	ms/cm^2^
$V_{0pNa}$	− 40	mV	$\tau _{1}$	10	h
$s_{Na}=s_{K}$	0.25	mV^−1^	$\tau _{2}$	10	h
$s_{pNa}$	0.09	mV^−1^	$v_{sp}$	0.5	nMh^−1^
$\eta$	0.012	cm^2^/μA	$v_{sc}$	0.25	nMh^−1^
$\xi$	0.17	$\cdots$	$k_{dp}$	0.5	h^−1^
$\tau _{K}$	0.000875	h	$k_{dc}$	0.5	h^−1^
$\tau _{pNa}$	0.00425	h	$K_{1}$	0.3	nM
$\tau _{K,Ca}$	0.00875	h	$K_{2}$	0.1	nM
$\alpha =\beta$	4	$\cdots$	$E_{syn}$	50	mV
$g_{dclock}=g_{per}$	0.05	ms/cm^2^	–	–	–

The parameter values are very similar as proposed in an earlier preliminary study^23,34^.

To quantify the amount of phase synchronization in a neural population, we assign an instantaneous phase to each neuron as in^39^

8 $$\begin{aligned} \phi _{i}(t)=2\pi \frac{t-t^{m}_{i}}{t^{m+1}_{i}-t^{m}_{i}} \end{aligned}$$

where $t^{m}_{i}$ is the instant of *m*-th spike of neuron *i*. Then we use two global instantaneous order parameters *S*^13^ and the Kuramoto order parameter^40^ as follows:

9 $$\begin{aligned} S(t)=\frac{2}{N(N-1)}\sum _{i\ne j}\cos ^{2} \left( \frac{\phi _{i}(t)-\phi _{j}(t)}{2}\right) \end{aligned}$$

The global order parameter *S* is the long-time-average of *S*(*t*) at the stationary state and measures collective phase synchronization in oscillations of membrane potentials of all neurons, viz $S = <S(t)>_{t}$. *S* is bounded between 0.5 and 1. If neurons spike out-of-phase, then $S\simeq 0.5$ where they spike completely in-phase $S\simeq 1$. For states with partial synchrony $0.5< S < 1$.

We have also calculated the more commonly used the Kuramoto order parameter^40^:

10 $$\begin{aligned} R(t)e^{i\theta }=\frac{1}{N}\sum _{j}e^{i\phi _{j}(t)} \end{aligned}$$

with $R = <R(t)>_{t}$ where $0 \le R \le 1$. $R = 0$ indicates asynchronous, while $R = 1$ completely synchronous, oscillations.

We also used a generalized susceptibility as the relative root-mean-square fluctuations in the given order parameter^14^:

11 $$\begin{aligned}&\kappa _{S}=\left( \frac{<S^{2}(t)>-<S(t)>^{2}}{<S(t)>^{2}}\right)^{1/2} \end{aligned}$$

12 $$\begin{aligned}&\kappa _R=\left( \frac{<R^{2}(t)>-<R(t)>^{2}}{<R(t)>^{2}}\right)^{1/2} \end{aligned}$$

We have carefully studied the synchronization transition of *N*-coupled neurons with gap junction coupling under the control of sleep-related biological drives. To clearly observe the difference between sleep and wake, we adjusted the sleep-related biological drives to keep neurons only in a sleep (external input $I_{dclock_{freei}}=0$) or wake ($I_{dclock_{freei}}=2.5$) state. First, the topology of the network is determined by the elements of the adjacency matrix $ADJ=(a_{ij})_{N\times N}$. The values of these elements $a_{ij}$ are 1 or 0, which satisfies $a_{ij}=1$ if node *i* and node *j* are connected; or else, $a_{ij}=0$. Since neurons are connected to each other by electrical coupling, the adjacency matrix is symmetric and $a_{ii}=0$. The coupling strength *g* is changeable as an independent variable to observe synchronization transition. We then integrate Eqs. (1)–(14) using fourth order Runge-Kutta method with a fixed time step $\bigtriangleup t = 10^{-4}$ h. A spike is detected when *V* crosses the threshold of $-20$ mV in a positive direction. Finally we obtain the phase of all neurons and calculate *S*(*t*) and *R*(*t*) at every time instant. We make observations after 48 h to ensure the validity of the results. For each trial, initial conditions are randomly and independently selected for all neurons with uniform probability from $-10$ to 80 mV for the membrane voltage variable $V_{i}$. Then, the results of each experiment are repeated 10 times and the final results are obtained after taking the average. Due to the different coupling strengths required for sleep and wake, we take a step size $\Delta g=10^{-4}$ during sleep and $\Delta g=10^{-3}$ during wake. The results are reported for $N=100$. The model was coded in Matlab 2019b.

## Results

### Regular networks

The first structure that we consider is a regular network consisting of a two-dimensional lattice of side $L (N = L \times L)$ with $L=10$. Each neuron is coupled to the upper, lower, left, and right neurons (the degree of each neuron is 4, $k=4$). The results in the sleep and wake states are shown in Fig.1. During sleep, in Fig.1a, it is observed that *S* and *R* does not vary monotonically with increasing *g*, but reveals a fluctuating behavior. At $g=0.001$ (orange), the neurons almost reach synchronization ($S/R \simeq 1$), however, this phenomenon changes drastically, and the neurons become almost completely out-of-phase as *g* increases to 0.003 (green, $S \simeq 0.52, R \simeq 0.22$). It is a bit surprising as one would expect a monotonic synchronization as the increase of *g*. This fluctuating behavior is called the frustrated synchronization. This phenomenon has been reported for the Kuramoto model on human connectome network which has a hierarchical modular structure^41^ and Hodgkin-Huxley network models of spiking neurons in gamma-band which has a hierarchical modular network^14^. The common point of these results is that the structure of the network is hierarchical modular network and research believes that the cause of this phenomenon is a manifestation of hierarchical modular structure of the underlying network. Nevertheless, the frustrated synchronization appears under the regular network. To further study the dynamics of neurons, we have hence researched the raster plots of all neurons under different coupling strengths *g*. Such raster plots for four values of *g* are shown in Fig. 1b where *g* is 0 (red), *g* is 0.001 (orange), *g* is 0.003 (green), and *g* is 0.01 (cyan). It is not difficult to find that the neurons in the network are first in-phase, then out-of-phase, and finally in-phase again with increases in *g*. Raster plots further verify a fluctuating behavior (frustrated synchronization).

During wake, it is observed that the network shows an overall discontinuous transition from asynchronization to synchronization in Fig. 1c. This discontinuous transition is not the same as the frustrated synchronization (during sleep), and this type of synchronization presents a consistent mechanism with those seen for phase oscillators in heterogeneous networks^42–44^, namely, explosive synchronization. As is shown in Fig. 1d, before the explosive synchronization occurs, the network is basically still in a completely disordered phase. See $g = 0.033$ (dark green) and $g = 0.034$ (magenta) raster plots which are just before and after the explosive synchronization transition point. This shows that the network has indeed undergone a sudden change from the disordered phase to the ordered phase. In a previous work^24,25,45^, we judged wake or sleep on the basis of the local field potential (LFP). Numerous previous researches have shown that the sleep for Drosophila link with reduced LFP activity compared to wake, moreover the authors found a transitional sleep stage associated with a 7–10 Hz oscillation during spontaneous sleep in flies and this oscillation is largely absent during wake^20^. Therefore, the frequency of network oscillations is different during sleep and wake. We can speculate that the frequency may be the key element in influencing the type and shape of synchronization transition curves. Hence, the type of synchronization transition is different between sleep and wake in the same network structure. In addition, it is evident that synchronization is easier to achieve during sleep than wake because sleep requires only a small coupling strength *g* to achieve synchronization. This is because the LFP has an irregular or a regular spike (chaotic state) during wake, however, the LFP becomes regular bursting during sleep. We can also see from Fig. 1 that qualitatively similar results of the synchronization diagrams are acquired whether we select *S* or *R*, except for the more refined statistics provided by *S* which enables us to determine the transition point clearly. Hence, we demonstrate the results only based on the order parameter *S* for the rest of this paper.

**Figure 2:**
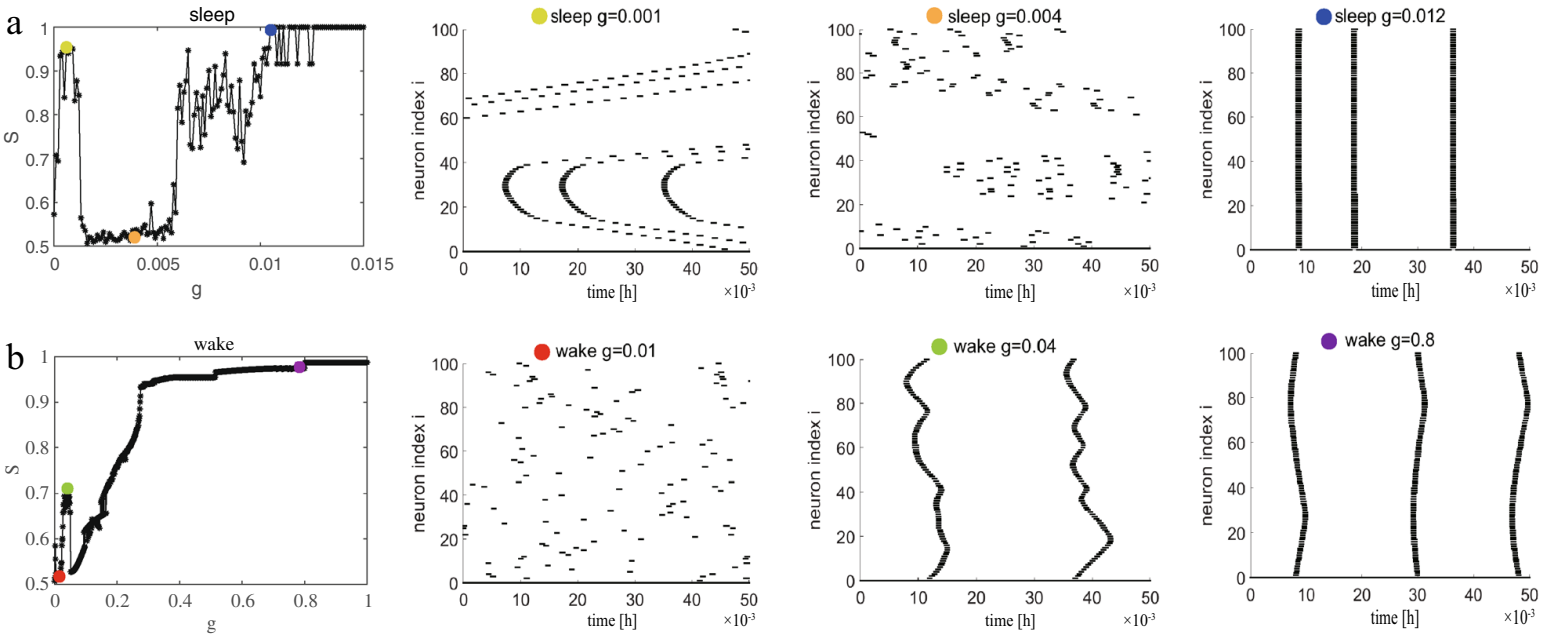
Synchronization diagrams of the coupled neurons in ring-shaped regular network with ($k=4$) during sleep and wake. (**a**) The global order parameter *S* and corresponding raster plots during sleep. (**b**) The global order parameter *S* and corresponding raster plots during wake.

For the sake of comparison, we also analyze another regular network, a ring-shaped regular network where each node also has 4 neighbors ($k=4$). Synchronization diagrams in sleep and wake states for coupled neurons are illustrated in Fig. 2. During sleep, frustrated synchronization is observed, which is similar to Fig.2, whereas, the fluctuating behavior is more exaggerated compared with Fig. 1 and the raster plots are different. At $g=0.001$ (yellow green) in Fig. 2a, the neurons achieve special synchronization, which is different from Fig. 1b (orange, complete synchronization) and appears to be the quasi periodicity synchronization^46–48^ since neuronal interactions are local, there are no long-range connections in ring-shaped regular network. Therefore, increasing synaptic strength *g* cannot eliminate phase lags among neurons coming from remote areas of the network, and leading to the formation of wave like pattern in order of neuronal spikes. On the basis of many previous studies on the synchronization of neural networks, especially the HH neural network, this similar phenomenon has been introduced in^13,14^, Khoshkhou and Montakhab observed that HH neurons reach quasi periodic partial synchronization in regular network. However, the increasing *g* does not lead to a transition in regular network. Wang et al.^49^ investigated the effects of information transmission delay for the synchronization transition on small-world networks and showed that short delays induce zigzag fronts of excitations, whereas longer delay lengths the synchrony of excitations in the network can again be enhanced due to the emergence of in-phase synchronization. That is, quasi periodicity synchronization does not disappear as the coupling strength increases. It is interesting to note that we not only discovered the quasi periodicity synchronization in a ring-shaped regular network but also that the network can finally realize complete synchronization at $g=0.012$ (navy blue).

Then, we are curious whether similar phenomena will occur during wake. As predicted, we discover the quasi periodicity synchronization during wake in raster plots. In Fig. 2b, it seems that a discontinuous transition has taken place but it is not. The raster plots prove that the network is a continuous transition. What is more, different from sleep period, the quasi periodicity synchronization state appears from $g\simeq 0.04$ and keeps robust when *g* is increased further as the perspective of raster plots is the same in quality from $g = 0.04$ to $g = 1.00$. We cannot increase *g* to arbitrary large values, as after a certain value (near $g \simeq 1.00$) neurons start subthreshold oscillations without spike generation^50^. We therefore conclude that neurons in ring-shaped network with purely local interactions lead to local order. Moreover, when the neural network has no random long-range connections, the network can finally achieve synchronization with the increase of *g* during sleep but not during wake. A Comparison between Figs. 1 and 2 shows that both the synchronization diagrams ($S-g$) and the raster plots are different. It might have been inferred that the structure of the homogeneous lattice network ($k=4$) in Fig. 1, the way neurons are connected, would lead to a different synchronization transition pattern from that of homogeneous ring-shaped network ($k=4$), that is to say the influence of the long-range connections may change this phenomena.

According to the results associated with Figs. 1 and 2, we can draw the following conclusions: (1) The type and shape of transition curves ($S/R - g$ plots) are different between sleep and wake, which may be due to the difference in frequency, especially, sleep requires only a small coupling strength *g* to achieve synchronization. (2) The network topology structure can affect the synchronization transition pattern and the synchronization type of neurons (raster plots).

### Networks with random long-range connections

**Figure 3:**
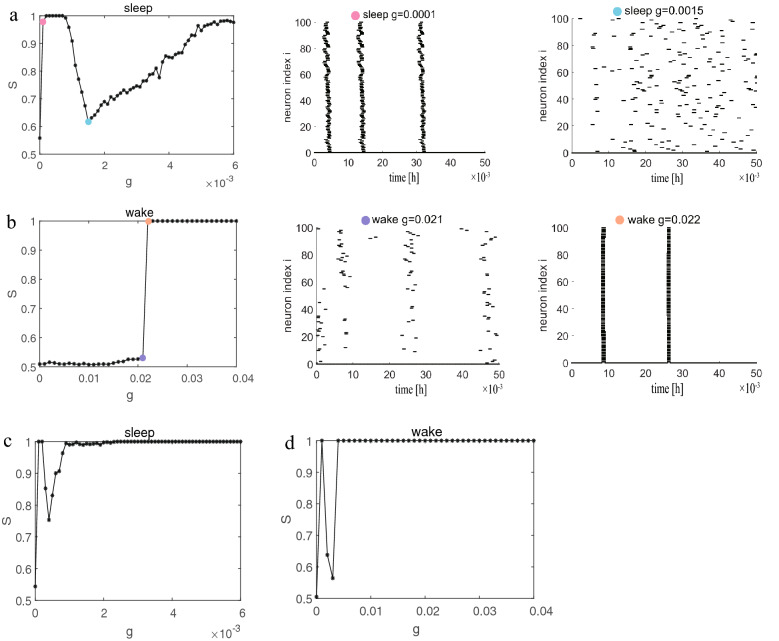
The lattice network with random long-range connections. (**a**)–(**b**) Each neuron randomly adds 1 long-range connection during sleep and wake. (**c**)–(**d**) Each neuron randomly adds 10 long-range connections during sleep and wake.

Based on the analysis in the previous subsection, we next discuss the role of random long-range connections, that is, adding random long-range connections to the regular network. First, two random networks where each neuron is randomly increased by 1 or 10 long-range connections based on the lattice network are constructed. We note that the synchronization results shown in Fig. 3 are very similar to those in Fig. 1. The little difference is that only requires a smaller coupling strength to achieve synchronization than the lattice network during sleep. It is not difficult to understand that this is because each node adds a long-range connection to make the network denser and easier to synchronize. Similarly, explosive synchronization occurs during wake see $g = 0.021$ (modena) and $g = 0.022$ (bright orange) raster plots in Fig. 3b. So we wondered whether we would get the similar results (less coupling strength required to achieve synchronization) when each neuron randomly adds 10 long-range connections. The result is shown in Fig. 3c–d. The answer is no doubt and even when the coupling strength is 0.0004 (the lowest point of *S*) in Fig. 3c, the network is almost synchronized. But, during wake, the synchronization diagram seems to be different from all previous phenomena in Fig. 3d. The obtained results are incredible. A synchronous regime (the frustrated synchronization patterns) similar to synchronization during sleep is observed for the first time, where *S* does not vary monotonically with increasing *g*. Moreover, explosive synchronization still occurs in Fig. 3d. Next, the random long-range connections are increased to 20 (not shown). The obtained results during sleep do not change much. During wake, the slightly increase of *g* makes the network completely synchronized. These results indicate that random long-distance connections have little effect on the synchronization transition during sleep and only accelerate synchronization, but have a significant impact on wake.

**Figure 4:**
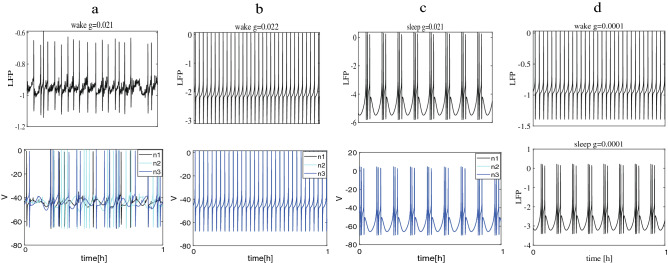
The LFP of two random networks and membrane potential *V* of neurons. (**a**)–(**c**) The network structure is each neuron in the lattice network randomly add 1 long-range connections. (d) The number of random long-range connections is 10. (**a**) and (**b**) The LFP plots and corresponding membrane potential plots at $g=0.021$ and $g=0.022$ which corresponds to Fig. 3b (modena and bright orange) during wake. (**c**) The LFP plots and corresponding membrane potential plots at $g=0.021$ during sleep. (**d**) The LFP plots for $g=0.0001$ during sleep (bottom) and wake (top).

To seek the underlying reason of this phenomenon, we observed the LFP of these two random networks and membrane potential *V* of neurons for different values of *g* during wake and sleep. The LFP is considered as an important reference indicator for the networks^24,25,45^. The LFP is calculated by the distance-dependent LFP^24^. $LFP=\sum _{\varepsilon =1}^{N} V_{\varepsilon }Shape(r_{\varepsilon })$, when $r_\varepsilon <\alpha$, $Shape(r_\varepsilon )=1$, or else $Shape(r_\varepsilon )=\alpha ^{m} r^{-m}_{\varepsilon }$, where $r_\varepsilon$ means the distance from the neuron to the electrode, $Shape(r_{\varepsilon })$ means the single neuron shape function, $\alpha$ is the cutoff distance to avoid a singularity, *m* is a decay exponent. The obtained results are shown in Fig. 4. First, the explosive synchronization of Fig. 3b during wake at $g = 0.021$ (modena) and $g = 0.022$ (bright orange) again is considered by the LFP and membrane potential *V* in Fig. 4a–b. We find that the LFP is in a chaotic state at $g=0.021$ but becomes a regular spike at $g=0.022$. The membrane potential *V* is also observed by randomly selecting three neurons (*n*1, *n*2, *n*3). It is obvious that contrary to the results obtained at $g = 0.022$ (see Fig. 4b, bottom), the three neurons spike out-of-order at $g = 0.021$ (see Fig. 4a,bottom). By analyzing the LFP and membrane potential *V*, it proves once again that the network has indeed undergone a sudden change from the disordered phase to the ordered phase. Then, to explore the difference between 10 long-range connections and 1 long-range connection, we compared the LFP of the two network structures. In Fig. 4d, the LFP of the network where each neuron randomly adds 10 random long-range connections are drawn at $g=0.0001$. It is interesting to note that we find that even though the coupling strength is smaller, the LFP in Fig. 4d is more regular than Fig. 4a during wake by comparing Fig. 4a ($g=0.021$, top) and Fig. 4d ($g=0.0001$, top). In other words, due to the denser long-range connections, the network has reached synchronization with a low increase *g*. Hence, we speculate that the network realizes synchronization rapidly and only a small coupling strength is required due to link density. Finally, why it is easier to achieve synchronization during sleep than during wake is explained. Regardless of coupling strength *g* and network structure, the LFP manifests more regular bursting as well as the spikes of neurons are more aligned during sleep than during wake by comparing Fig. 4a and c ($g=0.021$).

**Figure 5:**
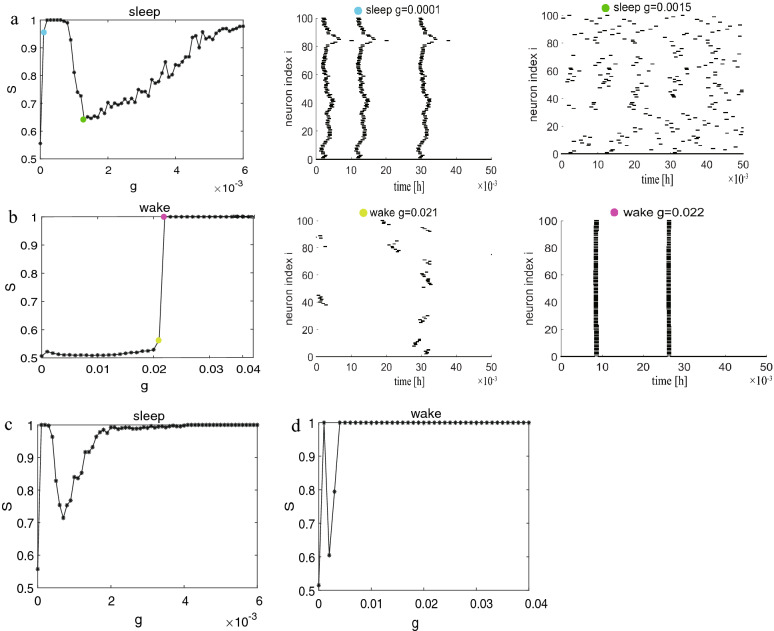
The ring-shaped regular network with random long-range connections. (**a**)–(**b**) Each neuron randomly adds 1 long-range connection during sleep and wake. (**c**)–(**d**) Each neuron randomly adds 10 long-range connections during sleep and wake.

Similarly, we add as many random long-range connections to the ring-shaped regular network as we do to the lattice network. Dependence of order parameter *S* on coupling strength *g* for two ring-shaped networks where each neuron randomly adds 1 or 10 long-range connections are illustrated in Fig. 5. This evolution process in Fig. 5a are almost the same as that of a ring-shaped regular network in Fig. 2a. The raster plots are also different from Fig. 3a. For example, at $g=0.0001$ (sky blue), the S-shaped wave pattern appears again, which means neurons reach quasi periodicity synchronization. During wake, it is seen that the results obtained in Fig. 5b are clearly different from Fig. 2b but still similar to Fig. 3b. Because the difference between Figs. 5 and 2 is whether there is the random long-range connection, and the difference from Fig. 3 is that the shape of network is different. So this interesting results indicate that random long-range connections (and not the shape of the network) are the main topological factor that plays an important role in the resulting synchronization transition during wake. This conclusion is consistent with that in lattice network.

### Effects of link density and long-range connections

**Figure 6:**
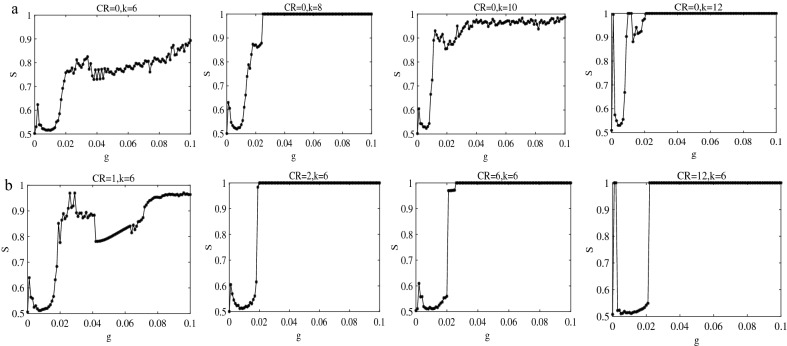
Effects of link density and long-range connections during wake. (**a**) The ring-shaped regular network with different edge density (from left to right is $k=6, k=8, k=10, k=12$). (**b**) The ring-shaped network with different long-range connections (the connection distance from left to right is $CR=1, CR=2, CR=6, CR=12$).

By comparing Figs. 3 and 5, we acquire an conclusion that no matter whether the shape of the network is circular or lattice, the qualitatively similar results are obtained after adding random long-range connections. Especially, the synchronization transition during wake has changed dramatically compared to Fig. 2. We note that one might suspect that the results are found to be probably due to the increase of link density *k* rather than the effect of random long-range connections or connection distance *CR*. Hence, to test the effect of link density *k*, we fix the coupling strength and only increase the degree of each node in ring-shaped regular network to see the order parameter *S*. The obtained results show that as the network becomes denser and denser, the network becomes easier to synchronize. However, we find that the value of *S* is similar under different *g* (not shown). The different link densities *k* for each node are selected to draw detailed transition curves ($S - g$ plots) in Fig. 6a. We observe that the synchronization transition is similar to $k=4$ in Fig. 2b until the link density of each node is increased to 12 ($k=12$). When $CR=0,k=12$, the frustrated synchronization and explosive synchronization appear at the same time again, because when the number of neighbors connected by a node continues to increase, it actually includes long-range connections. Combining Figs. 3d and 5d, we infer that this phenomenon (frustrated and explosive occur simultaneously) may be caused by a combination of link density and long-range connections.

Additionally, to further verify the role of long-range connections, we also only change the connection distance *CR*. A ring-shaped regular network where each node has 4 neighbors ($k=4$) is established, and two edges are added symmetrically at a distance of *CR* on this basis. That is to say, the average degree of each node in the network is unchanged $k=6$, and the only change is the connection distance between the two edges added and the node. For example, the node 1 is connected to nodes 2 and 3 and nodes 100 and 99. If the connection distance is $CR=0$, then the two symmetrical edges added are node 4 and node 98 (this situation is exactly the same as Fig. 6a $CR=0,k=6$). If the connection distance is $CR=1$, then the two symmetrical edges added are node 5 and node 97. As the connection distance becomes larger, it is obvious that the value of *S* gradually becomes different between different *g* (not shown). We also choose some different connection distances *CR* to draw detailed transition curves in Fig. 6b. It is worth noting that when the connection distance *CR* increases, the synchronization transition starts to become different and turns into the explosive synchronization. Furthermore, we discover that when the connection distance *CR* increases from 1 to 2, the synchronization transition begins to explosive synchronization and remains unchanged until $CR=42$. It can be proven that random long-range connections play an important role in influencing synchronization transitions during wake.

### Small-world networks

**Figure 7:**
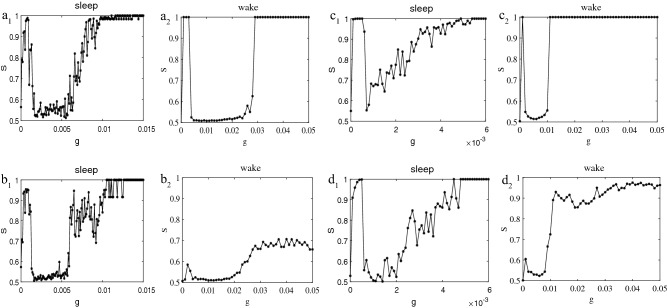
Synchronization comparison diagrams for the WS network (*p* = 0.1) and ring-shaped regular network during sleep and wake. (**a**) and (**c**) $S-g$ curves for the WS network with $k=4, p=0.1$ and $k=10, p=0.1$. (**b**) and (**d**) ring-shaped regular network with $k=4, p=0$ and $k=10, p=0$.

To further verify the role of random long-range connections, we investigate Watts-Strogatz (WS) networks which have high clustering coefficient, as well as short average path length. As a matter of fact, the WS network is the closest to the synaptic connections among neurons in the brain from the point of view of neuroscience since it has been reported that the brain networks at the microscopic level are similar to WS networks^51^. We construct the WS networks where the average degree of each neuron is also 4 ($k=4$) and 10 ($k=10$) respectively by random rewiring of ten percent of links of a regular ring $(p=0.1)$. The results are shown in Fig. 7. For a better comparison, the corresponding synchronization plots for the ring-shaped regular network with $k=4$ (see Fig. 7b) and $k=10$ (see Fig. 7d) during sleep and wake are given. During sleep, regardless of the average degree of each neuron is 4 or 10, the synchronization curves $S-g$ in Fig. 7$a_{1}$ and $c_{1}$ are all similar to the Fig. 7$b_{1}$ and $d_{1}$. From this we conclude that the synchronization transition will not change due to different structures during sleep. Investigation of raster plots of the network with $k=4$ during sleep reveals that when synaptic interaction is weak, neuron spikes still demonstrate wave patterns. However, the effect of random long-range connections in the case of WS network is also important, increasing *g* will eventually lead to interactions among various parts of the network which eventually leads to complete synchronization at $g=0.01$. A similar process also occurs in the WS network with $k=10$ and the difference is that the magnitude of the *g* is smaller (not shown).

During wake, different from the ring-shaped regular network with $k=4$ in Fig. 7$b_{2}$ and $k=10$ in Fig. 7$d_{2}$, the explosive and frustrated synchronization is observed in the WS network which is consistent with that in Fig. 5d. This result again confirms the inference of the previous subsection. We also discover that as the average degree of neurons increases, the synchronization process $S-g$ is not exactly the same. In Fig. 7$c_{2}$, this explosive phenomenon occurs when the value of *g* changes from 0.01 to 0.011. However, after $g=0.028$, the network reaches synchronization $S\simeq 1$ in Fig. 7$a_{2}$. Based on the above discussion and analysis, the importance and necessity of the long-range connections have once again been proven. We can conclude that the synchronization transition will not change due to different structures and shapes of the network during sleep but the opposite is true during wake. Furthermore, the long-range connections are the critical factor affecting synchronization transition during wake.

**Figure 8:**
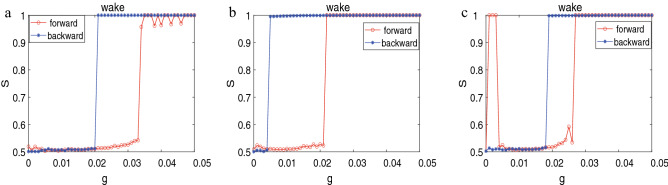
Transition to phase synchronization in forward and backward evolution of the networks. (**a**) The lattice network. (**b**) The ring-shaped regular network where each node randomly add 1 long-range connection. (**c**) The WS network ($k=4, p=0.1$).

In addition, the explosive synchronization is observed in the Figs. 1c, 3b, 5b and 7$a_{2}$ respectively. In previous reports, the phenomenon for explosive transition to phase synchronization, with a large hysteresis loop, was mentioned in the literature^13,14,44^. Boaretto et al.^44^ detected that the dynamical mechanisms for the onset and end of the bistability region of explosive synchronization. Moreover, in terms of a saddle-node bifurcation and a boundary crisis, the hysteretic behavior considering the forward and backward direction are given respectively. We perform a backward scan *g* from a highly synchronized state for several networks. The acquired results in Fig. 8. We also discover that the synchronization transition is accompanied by a hysteresis loop. The other networks researched here are qualitatively similar to that in Fig.8 (not shown).

## Conclusion

In a previous work, we researched the collective dynamics of neural networks with sleep-related biological drives in Drosophila. We demonstrated that the difference between sleep and wake is that the frequency and the spike pattern of neurons, as well as different time series of the LFP can be obtained under different network structures^25^. In this paper we have reported a systematic study of synchronization transition for neural networks with electrical synapses during sleep and wake. Our focus is to study the types of synchronization transitions during sleep and wake, and the influence of the topology and shape of the networks on the types of synchronization transitions that may occur. The mechanisms and patterns of synchronization transition we obtained during sleep are distinctly different from those we obtained during wake. The neural network during sleep generates an intermediate regime between order and disorder. Such a phenomenon has been previously shown to appear in the Kuramoto model on human connectome network which has a hierarchical modular structure^41^ and Hodgkin-Huxley network models of spiking neurons in gamma-band which has a hierarchical modular network^14^, however, our results indicate that such frustrated transition can also be observed during sleep, and it is furthermore independent of the structure and shape of the network. Moreover, as the network becomes dense, even if the synchronization $S-g$ is frustrated, we can also observe that the neurons are almost synchronized by the raster plots at the lowest point of *S* in Fig. 3$b_1$ and Fig. 5$b_1$. During wake, the synchronization transition of the network is very dependent on the network structure and three mechanisms of synchronization transition have emerged during wake: explosive synchronization, continuous synchronization and frustrated synchronization. On the other hand, from the perspective of the raster plots, a clear distinction occurs between sleep and wake. Whether sleep or wake, the synchronization types of neurons (quasi periodicity synchronization and unified synchronization) depend on shapes of the network (ring-shaped and lattice), in other words, neurons in a ring-shaped network appear quasi-periodic synchronization, but this phenomenon will disappear with the increase of random long-range connections. Interestingly, there is no need to increase long-range connections during sleep, and they can still achieve complete synchronization by the enhancement of synaptic interaction but not during wake, as shown in Fig. 2.

A large number of research results on the synchronization of HH (or HH-type) neurons have been reported in recent years^52–56^, however, much of such type of researches usually make use of phase oscillator models for instance the Kuramoto model^40^. A systematic study of synchronization transition is very necessary^13,14^. Khoshkhou et al. reported a systematic study of synchronization transition of HH neural network in beta-band and gamma-band. Based on the results of previous research, we know that there are obvious oscillations at approximately 8 Hz during sleep. Hence, this article also researches the synchronization transition in network models of spiking neurons in alpha-band. Here, our focus is on determining the types of phase transitions (e.g., continuous, explosive, frustrated) that may occur in a collection of neurons during sleep and wake, and how that may rely on underlying network structure. By comparing the research of HH neural networks in gamma-band and beta-band, we also discover that the mechanisms and patterns of synchronization transition are distinguishing. For example, in a one-dimensional and two-dimensional lattice, the authors found no transition for the network with electrical synaptic in the gamma-band and found a continuous transition in the beta-band, however, in the beta-band, the neurons are not fully synchronized as *g* increases, while here for the alpha-band we not only discovered the transition (discontinuous transition) whether in a lattice network or a ring network but also the network could finally realize complete synchronization. Furthermore, we also discover that discontinuous transitions (explosive synchronization, explosive and frustrated synchronization occur at the same time) occur in the WS network ($p=0.1$), while explosive synchronization (a smooth transition) in gamma-band (beta-band) occurs in such networks with electrical synapses.

## Data Availability

All data generated and analysed in the manuscript are reproducible based on the algorithms detailed in the article (see the “Methods” and the “Results” sections).
